# The Prognostic Significance of Secondary Mitral Regurgitation in Heart Failure Patients with Varying Estimated Pulmonary Artery Systolic Pressure

**DOI:** 10.31083/j.rcm2411316

**Published:** 2023-11-16

**Authors:** Roubai Pan, Yan Xu, Xiao Zong, Qian Yang, Xierenayi Tudi, Rui Xi, Qin Fan, Rong Tao

**Affiliations:** ^1^Department of Cardiovascular Medicine, Ruijin Hospital, Shanghai Jiao Tong University School of Medicine, 200025 Shanghai, China

**Keywords:** heart failure, pulmonary hypertension, ePASP, pulmonary artery systolic pressure, secondary mitral regurgitation

## Abstract

**Background:**

Limited research has been conducted to investigate the impact of secondary mitral regurgitation (MR) in heart failure (HF) patients with different levels of estimated pulmonary artery systolic pressure (ePASP).

**Methods:**

A total of 468 patients suffering from HF and secondary MR were enrolled and categorized into non-severe and severe MR groups based on the degree of MR. The primary endpoint of the study was a composite of cardiovascular death and a first-heart-failure hospitalization. The secondary endpoints were the primary outcomes, individually. The outcomes of the two groups were compared. Patients were further classified based on whether their ePASP was ≥50 mmHg or <50 mmHg. Subsequently, the outcomes of the non-severe and severe MR groups were compared within each ePASP category.

**Results:**

In a median (SD) follow-up of 694 (410) days, severe MR was associated with higher risk for primary endpoints in patients with heart failure, especially in those with ePASP ≥50 mmHg. In patients with ePASP <50 mmHg, the prognostic value of severe MR was diminished.

**Conclusions:**

Assessment of the severity of MR can identify heart failure patients who are at greater risks for poor clinical outcomes. Additionally, the prognostic value of secondary MR was more pronounced in patients with elevated ePASP.

## 1. Introduction

Pulmonary hypertension (PH) is a common consequence of hemodynamic disturbances in heart failure (HF), with a prevalence of 60%–70% in systolic HF patients and 50% in diastolic HF patients who undergo right-sided heart catheterization [1]. PH is associated with a higher symptoms burden, increased mortality, and hospitalization in HF patients [2, 3]. Furthermore, the chronic elevation of pulmonary artery systolic pressure (ePASP) produces right ventricular dysfunction and remodeling, which are the most predictive indicators for patients with HF [4]. The pathogenesis of PH begins in the earliest stage of HF, yet there is currently a lack of an appropriate therapeutic strategy for this particular subset of HF patients. Effective management of PH requires addressing its underlying cause to improve patient outcomes and prevent further deterioration.

Secondary mitral regurgitation (MR) is a valvular heart disorder characterized by inadequate coaptation of the mitral leaflets, resulting from left ventricular dysfunction caused by either ischemic or non-ischemic cardiomyopathies, in the absence of structural or degenerative abnormalities. As HF progresses, MR arises from localized or generalized remodeling of the left ventricle (LV) [5]. Impaired coaptation of mitral-valve leaflets results in volume overload of the left ventricle and an increase in diastolic wall stress, which affects LV remodeling. Patients with HF and secondary MR are at a higher risk for unfavorable cardiac outcomes compared to those without secondary MR [6]. In addition, secondary MR is a cause of increased pulmonary pulsatile loading, and a driver of PH in HF patients. MR frequently coexists with PH in patients with HF [7]. These conditions collectively contribute to volume overload on the overstressed heart and are believed to have detrimental effects on patients with HF.

The presence of PH is used as a hemodynamic criterion in patients with primary MR [8]. Even after mitral valve repair, higher ePASP is related to increased risk of death in patients with secondary MR [9]. Nevertheless, secondary MR in HF patients, particularly in those with varying levels of ePASP, has not been thoroughly investigated. The aim of the present study was to address the existing knowledge gap by investigating the impact of secondary MR severity on adverse outcomes in patients with HF and varying levels of ePASP.

## 2. Materials and Methods

### 2.1 Study Design and Sample

This prospective, single-center study was designed to determine the prognostic significance of secondary MR in HF patients with varying ePASP. We examined the records of 956 consecutive patients hospitalized with signs of HF at the Department of Cardiology, Ruijin Hospital, Shanghai Jiao Tong University of Medicine, China, from March 2017 to March 2020. Subsequently, the inclusion criteria were fulfilled by 468 patients: (1) patients with underlying heart disease that could lead to HF; (2) left ventricular dysfunction, defined as <50% left ventricular ejection fraction (LVEF); and (3) MR related to LV remodeling. Exclusion criteria were: (1) HF patients with intrinsic valvular deficits or prior valvular intervention, and (2) patients with noncardiac causes of PH; (3) patients with previous cardiac resynchronization therapy (CRT) or installation of implantable cardioverter-defibrillators (ICD). To exclude noncardiac causes, patients diagnosed with pulmonary arterial hypertension confirmed by right heart catheterization (RHC) either before hospitalization or during follow-up were excluded from the study cohort. Non-invasive methods such as arterial blood-gas measurement, pulmonary function tests, chest computed tomography (CT), or computed tomography pulmonary angiography were employed when there were suspicions of PH associated with lung disease or chronic thromboembolic pulmonary hypertension. The medical history was examined in order to exclude drug- and toxin-associated PH. Other biochemical, hematological, immunological, HIV, and thyroid-function tests were performed to identify other conditions related to PH. During the enrollment process, demographic data was collected, and baseline measurements including laboratory tests were conducted. Throughout the study duration, medications including renin-angiotensin-aldosterone system inhibitors and β-blockers were prescribed and titrated to reach optimal dosages for each individual with HF. During hospitalization, medication doses were gradually adjusted based on factors including blood pressure (BP), heart rates, symptoms, and laboratory test. The final dose adjustment during hospitalization occurred within 2 days before anticipated discharge. Following discharge, the patients were asked to record their daily heart rates and BP, and undergo laboratory tests. The doses of medication for HF were adjusted based on these assessments. Patients were instructed to have follow-up visits every 1–2 months during the first six months following discharge. Medication adherence was assessed through hospital visit or telephone. The follow-up interval for patients receiving optimal HF therapy was at least 3 months. CRT or ICD placement was performed during the follow-up period in accordance with current recommendations and patients’ consent [10]. Throughout the follow-up visits, a total of 6 patients underwent CRT and 12 patients had ICD installed.

The institutional review committee of Rui Jin Hospital, Shanghai Jiao Tong University of Medicine approved the study and confirmed that the research adhered to the ethical guidelines of the 1975 Declaration of Helsinki. Informed consent was obtained in writing from all participants prior to screening.

### 2.2 Echocardiographic Quantifications

Two-dimensional and Doppler transthoracic echocardiography imaging was conducted at baseline as a routine clinical practice. All patients included in the study were hospitalized due to signs and symptoms of HF and underwent echocardiography when they were hemodynamically stable. All echocardiographic examinations were conducted using a Vivid E9 imaging system with an M5S transducer (General Electric Medical Health, Milwaukee, WI, USA), and performed by skilled cardiac sonographers. All the echocardiograms were scrutinized by cardiologists with board certification. In the parasternal long-axis view, the LV end-diastolic dimension (LVEDD), the LV end-systolic dimension (LVESD), and the left atrial dimension (LAD) were measured using two-dimensional echocardiographic images. The vena contracta width (VCW) was measured at the narrowest portion of the regurgitant flow occurring at, or immediately downstream of the orifice, using a parasternal long-axis image with magnification. The Simpson biplane approach was applied to LVEF evaluation. Using continuous-wave Doppler echocardiography we measured the peak velocity of tricuspid regurgitation. The ePASP was determined with a simpliﬁed Bernoulli equation: $\text{ePASP}=4\times {\text{ peak tricuspid regurgitant velocity}}^{2}+\text{ mean right atrial pressure }$. The mean right atrial pressure was determined based on the diameter of the inferior vena cava and variations in response to respiration. MR was quantified using the proximal-isovelocity-surface area (PISA) technique. The effective regurgitant oriﬁce area (EROA) was obtained from the maximal PISA radius and the peak regurgitant velocity of a continuous-wave MR jet [11]. The stroke volumes were computed as the product of the time-velocity integral and the area of the annulus of each valve, using pulsed-wave Doppler methods. The regurgitant volume (RV) was computed by subtracting the stroke volumes of the mitral and aortic valves. The EROA, RV, and VCW were chosen as severity indices for MR. Severe MR was classified as an EROA ≥0.4 ${\mathrm{cm}}^{2}$, an RV >60 mL/beat, or a VCW ≥0.7 cm, whereas the non-severe MR group consisted of patients with echocardiographic indices below those levels [11]. VCW was used to classify the severity of tricuspid regurgitation (TR) in a semiquantitative manner. A VCW ≥0.7 cm was classified as severe TR, 0.3–0.69 cm as moderate TR, and <0.3 cm as mild TR. An ePASP of >50 mmHg was classified as ePASP elevation, because the value is thought to be connected to poorer results in patients with functional MR, and the prognostic effect of an ePASP of 50 mm Hg was also confirmed in our study [12].

### 2.3 Outcomes and Follow-Up

The primary endpoint of the trial was either cardiovascular disease-related mortality or hospitalization for HF. The secondary endpoints included individual cardiovascular disease and heart failure hospitalization (HFH). Cardiovascular mortality comprised death from decompensation of HF, acute HF, myocardial infarction, fatal arrhythmia, sudden cardiac death, and thromboembolism-related deaths. HF diagnosis was based on clinical symptoms, signs, abnormal test markers, and radiologic evidence. The results were collected by reviewing medical records and/or conducting telephone or hospital-based surveys. These results were confirmed by an independent group of clinicians.

### 2.4 Statistical Analysis

Patients were categorized based on the severity of MR: (a) non-severe MR group, and (b) severe MR group. A Kolmogorov-Smirnov test was performed for normal-distribution validation. Continuous variables in baseline characteristics were presented as the mean (SD) or median (interquartile range [IQR]) and were compared across subgroups using Student’s* t*-test or Wilcoxon rank-sum test, respectively. Numbers and proportions were presented for categorical variables. The Pearson chi-square test or the Fisher exact test was employed for comparison. The relationship between ePASP and the clinical outcomes was explored by using restricted cubic splines with four knots. LVEF, NT-proBNP (N-terminal pro–B-type natriuretic peptide), and LVEDD were presented as medians (IQR). We compared these three variables in the two subgroups with varying levels of ePASP with the Wilcoxon rank-sum test. The Kaplan-Meier method was utilized to describe event rates for the examination of time-to-first-event in each group with varied ePASP stages. Univariable Cox proportional hazards regression models were constructed to assess the probability of survival. The log-rank test was applied to the comparison of any two subgroups with distinct ePASP at each endpoint. Age, sex, body mass index (BMI), diabetes mellitus, hypertension, coronary artery disease, prior atrial fibrillation, hypercholesterolemia, anemia, estimated glomerular filtration rate (eGFR), and LVEF were adjusted in multivariable Cox proportional hazards regression models. The Schoenfeld residual test was used to verify the proportional hazards assumptions (**Supplementary Table 1**). Variance inflation factors were calculated for multicollinearity diagnosis (**Supplementary Table 1**). Participants who dropped from the study during the follow-up period were considered censored data. All *p* values were two-tailed, and *p* ≤ 0.05 was judged to be statistically significant. The R package 4.2.2 (https://cran.r-project.org/) was utilized for analysis.

## 3. Results

### 3.1 Baseline Characteristics

**Fig. 1.**
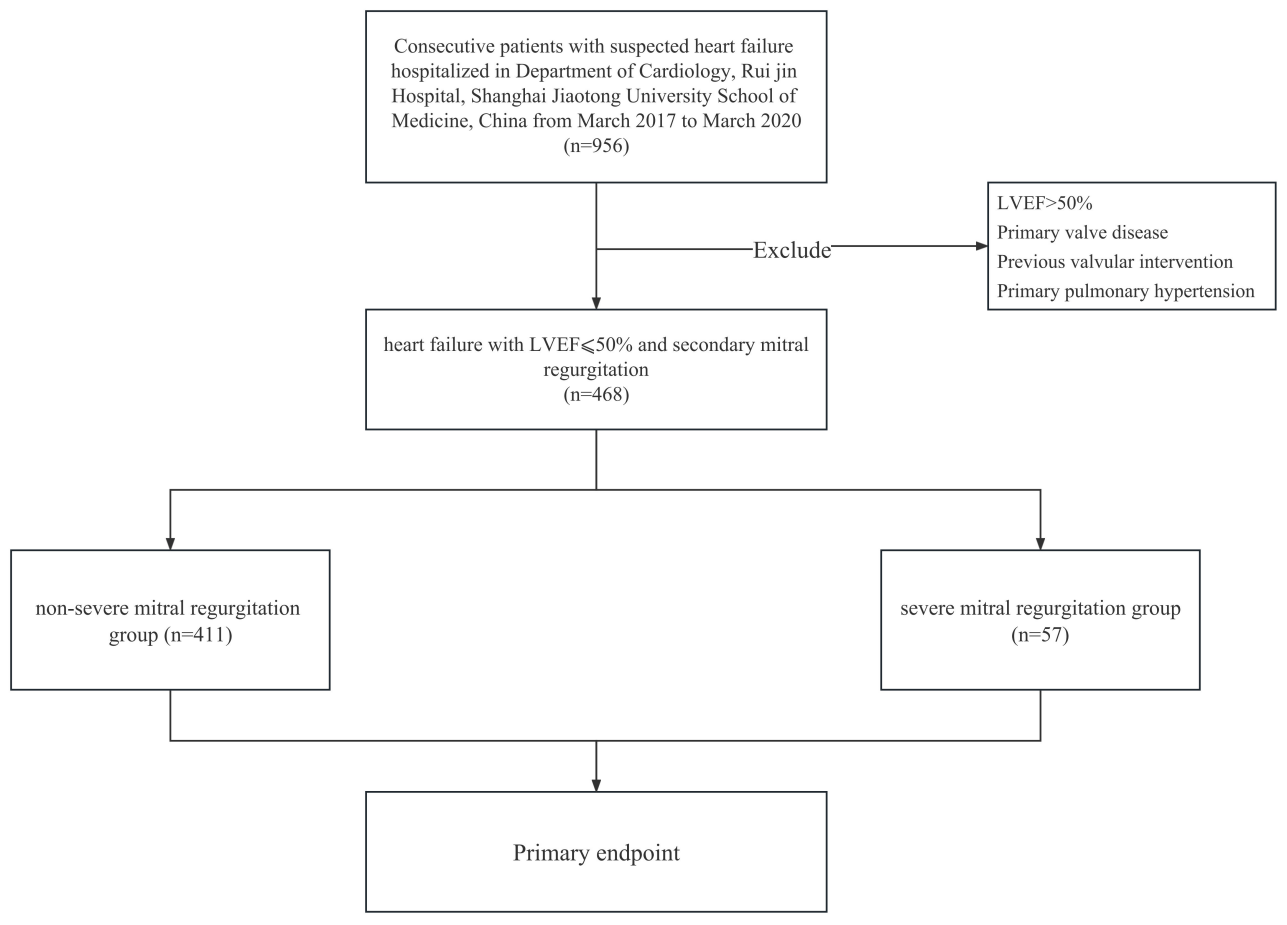
**Flow-chart of recruitment, grouping and follow-up of patients with heart failure**. LVEF, left ventricular ejection fraction.

The study included a total of 468 consecutive patients with a primary diagnosis of HF and a secondary diagnosis of MR. Among them, 57 of 468 patients (12.1%) were identified with severe MR based on echocardiographic assessment (Fig. 1). The mean age of the cohort was 62.5 years (SD 12.0), with males accounting for 83.5% (391) of the sample. The mean LVEF was 36.8% (8.5%).

Table 1 summarizes the baseline characteristics of the patients in the two subgroups. Patients with severe MR were more likely to be female, suffer from atrial fibrillation or atrial flutter and anemia, and were more likely treated with a diuretic and a mineralocorticoid-receptor antagonist (MRA). Furthermore, patients in the severe MR cohort had elevated levels of NT-proBNP and a reduced eGFR. Additionally, the majority of patients in the severe MR group had non-ischemic cardiomyopathy and were less likely to require percutaneous coronary intervention (PCI). Echocardiographic measurements revealed a higher ePASP, advanced structural enlargement, and LV dysfunction among those with severe MR. Moreover, the severe MR group demonstrated a higher incidence of TR.

**Table 1.** **Baseline characteristics of the study sample by degree of MR**.

			Non-severe MR	Severe MR	*p* value	Kolmogorov-Smirnov test *p*
			(*n *= 411)	(*n* = 57)		
Clinical						
	Female		61 (14.8%)	16 (28.1%)	0.02*	NA
	Age (median and IQR)		64 (55–71)	63 (55–69)	0.59	0.026*
	BMI, kg/$m^{2}$		25.1 (3.9)	23.2 (3.0)	<0.001***	0.185
	Diabetes		156 (38.0%)	21 (36.8%)	0.99	NA
	Hypertension		258 (62.8%)	31 (54.4%)	0.28	NA
	Hypercholesterolemia		63 (15.3%)	7 (12.3%)	0.68	NA
	History of atrial fibrillation or flutter		57 (13.9%)	17 (29.8%)	0.004**	NA
	Peripheral artery disease		55 (13.4%)	6 (10.5%)	0.69	NA
	Coronary artery disease		304 (74.0%)	28 (49.1%)	<0.001***	NA
	Prior PCI		245 (59.6%)	14 (24.6%)	<0.001***	NA
	Previous coronary artery bypass grafting		22 (5.4%)	1 (1.8%)	0.34	NA
	History of anemia		32 (7.8%)	5 (8.8%)	0.05	NA
	eGFR (mL/min/1.73 $m^{2}$)		73.2 (24.3)	66.2 (23.4)	0.04*	0.07
	Beta-blocker		360 (87.6%)	51 (89.5%)	0.84	NA
	ACEI or ARB or ARNI		330 (80.3%)	45 (78.9%)	0.95	NA
	MRA		219 (53.3%)	46 (80.7%)	<0.001***	NA
	Diuretics		187 (45.5%)	42 (73.7%)	<0.001***	NA
Related to HF						
	Reasons cardiomyopathy					NA
		Ischemic	313 (76.2%)	23 (40.4%)		
		Nonischemic	98 (23.8%)	34 (59.6%)	<0.001***	
	NYHA class					NA
		I	4 (1.0%)	0 (0%)		
		II	91 (46.5%)	17 (29.8%)		
		III	176 (42.8%)	34 (59.6%)		
		IV	40 (9.7%)	6 (10.5%)	0.08	
	NT-proBNP (median and IQR)		1229 (419–3234)	3391 (1816–6364)	<0.001***	<0.001***
Echocardiographic parameters						
	ePASP, mmHg (median and IQR)		35 (20–45)	46 (37–55)	<0.001***	<0.001***
	LVESD, mm (median and IQR)		48 (43–54)	57 (52–62)	<0.001***	0.018*
	LVEDD, mm (mean and SD)		61.2 (7.7)	68.1 (8.4)	<0.001***	0.054
	LAD, mm (median and IQR)		44 (40–48)	50 (47–54)	<0.001***	0.02*
	LVEF ≤40%		38 (30–45)	30 (27–34)	<0.001***	<0.01**
			236 (57.3%)	51 (76.0%)	< 0.001***	
	Tricuspid regurgitation					NA
		None	250 (60.8%)	14 (24.6%)		
		Mild	110 (26.8%)	18 (31.6%)		
		Moderate	55 (13.4%)	21 (36.8%)		
		Severe	6 (10.5%)	4 (7.0%)	<0.001***	

Variables are mean (SD), n (%), or median (IQR); * = *p* < 0.05; ** =* p* < 0.01; *** = *p* < 0.001. ACEI, angiotensin-converting enzyme inhibitor; ARB, angiotensin receptor blocker; ARNI, Angiotensin receptor/neprilysin dual inhibitors; BMI, body mass index; eGFR, estimated glomerular filtration rate; ePASP, estimated pulmonary artery systolic pressure; HF, heart failure; IQR, inter quartile range; LAD, left atrial dimension; LVEDD, left ventricle end-diastolic dimension; LVEF, left ventricular ejection fraction; LVESD, left ventricular end-systolic dimension; MRA, mineralocorticoid-receptor antagonist; NT-proBNP, N-terminal pro- B-type natriuretic peptide; NYHA, New York heart association; PCI, percutaneous coronary intervention; MR, mitral regurgitation; HF, heart failure; NA, not available.

### 3.2 Relationship between ePASP as a Continuous Variable and the Risk for Clinical Outcomes in Study Sample

**Fig. 2.**
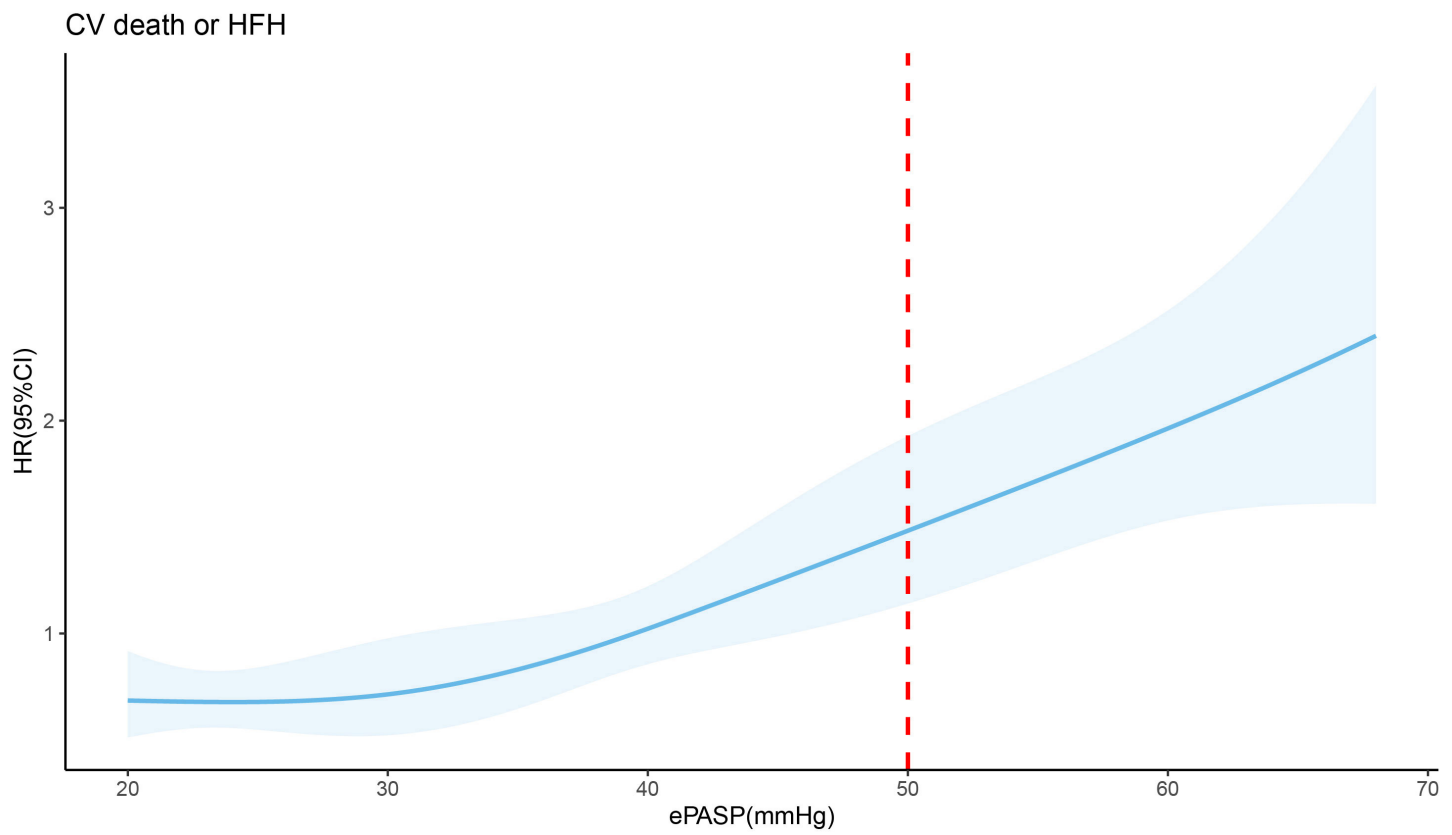
**Unadjusted relationship between a continuous ePASP and the hazard ratios for cardiovascular death or heart failure hospitalization in the study sample**. Shades areas show the 95% CI for the HR at every single pressure level. The dashed red line represents an ePASP of 50 mmHg. ePASP, estimated pulmonary artery systolic pressure; CV, cardiovascular; HFH, heart failure hospitalization; HR, heart rates.

A linear relationship between ePASP and risks for cardiovascular death or heart failure hospitalization is visualized in Fig. 2 (*p* for non-linearity = 0.64). Every 10 mmHg increase in ePASP is associated with a 32.3% increase in the risk for primary outcomes (hazard ratio [HR]: 1.32; 95% confidence interval [CI] = 1.19–1.47; *p* < 0.001). In an ePASP of 50 mmHg, patients in the study sample are at elevated risk for cardiovascular death or heart failure hospitalization (HR: 1.49; 95% CI =1.15–1.94, *p* = 0.003).

### 3.3 Correlation of LV Function and ePASP in Non-Severe and Severe MR Groups

**Fig. 3.**
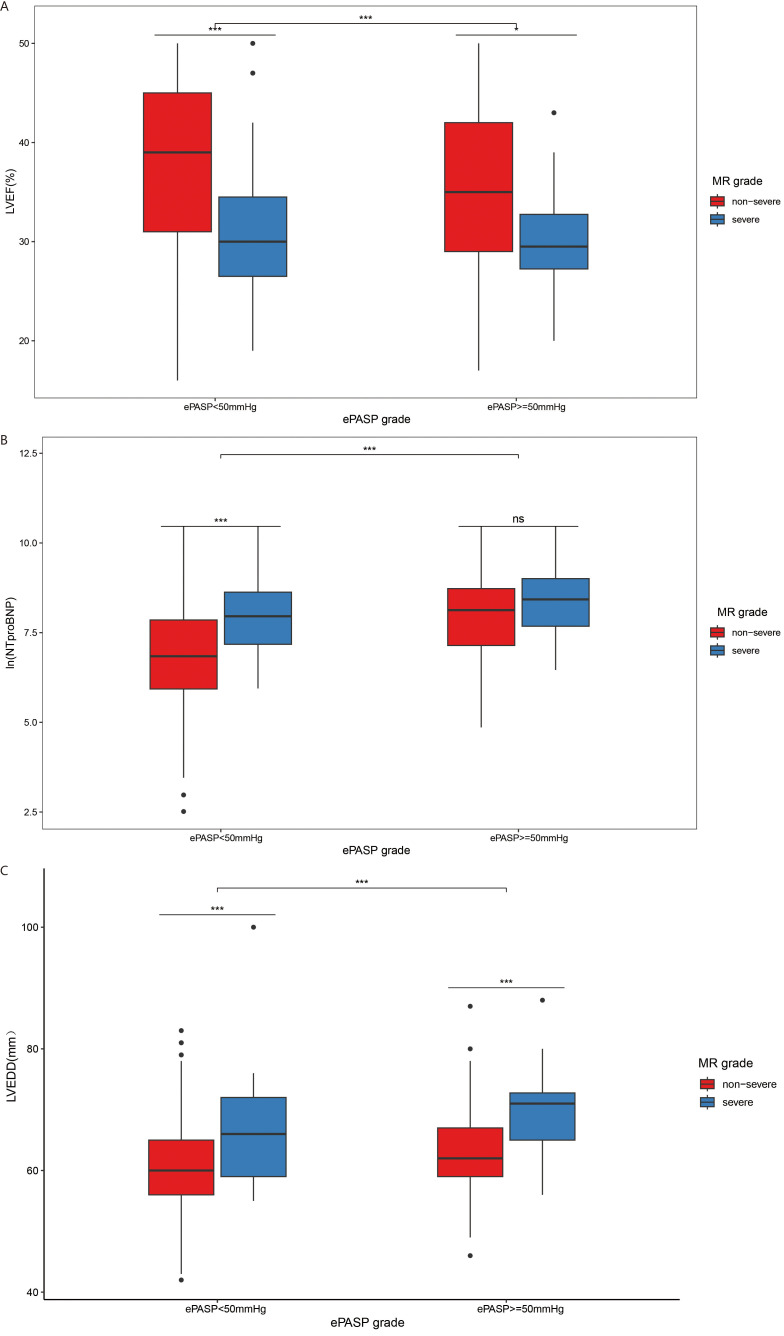
**The relationship between ePASP and LV function in non-severe and severe MR group**. Boxplots for (A) LVEF, (B) logarithmic form of NT-proBNP, and (C) LVEDD, comparing patients with ePASP <50 mmHg and ePASP ≥50 mmHg in the non-severe and severe MR groups. ns, not significant; * = *p <* 0.05; *** *p <* 0.001. LVEF, left ventricular ejection fraction; MR, mitral regurgitation; ePASP, estimated pulmonary artery systolic pressure; NTproBNP, N-terminal pro- B-type natriuretic peptide; LVEDD, left ventricle end-diastolic dimension; LV, left ventricle.

In the current study, a total of 100 patients (21.4% of the entire cohort) exhibited an ePASP measurement above 50 mmHg. Patients with ePASP ≥50 mmHg exhibited more severe LV dysfunction, as evidenced by LVEF (LVEF median [IQR]: 32.5% [IQR = 28%–40%] *vs* 38% [IQR = 30%–45%], *p <* 0.001) (Fig. 3A), NT-proBNP (logarithmic form of NT-proBNP: 8.24 [IQR = 7–9] *vs* 6.98 [IQR = 8–9], *p <* 0.001) (Fig. 3B), and LVEDD (LVEDD: 64 [IQR = 60–70] mm *vs* 61 [IQR = 57–66] mm, *p <* 0.001) (Fig. 3C). Among patients with a ePASP ˂50 mmHg, those with severe MR exhibited poorer LVEF (35% [IQR = 29%–42%] *vs* 39% [IQR = 31%–45%], *p <* 0.001), higher NT-proBNP (logarithmic form: 8.13 [IQR = 7–9] *vs* 6.84 [IQR = 6–8],* p <* 0.001) and greater LVEDD (62 [IQR = 59–67] mm *vs* 60 [IQR = 56–65] mm, *p <* 0.001) (Fig. 3). Similarly, severe MR was related to lower LVEF (29.5% [IQR = 27%–33%] *vs* 30% [IQR = 26%–34%],* p *= 0.01) and greater LVEDD (71 [IQR = 65–73] mm *vs* 66 [IQR = 59–72] mm, *p* < 0.001) among those with higher ePASP, but there was no substantial difference in NT-proBNP between non-severe and severe MR groups (logarithmic form: 8.43 [IQR = 8–9] *vs* 7.96 [IQR = 7–9], *p *= 0.18) (Fig. 3).

### 3.4 Clinical Outcomes Based on MR Severity

**Fig. 4.**
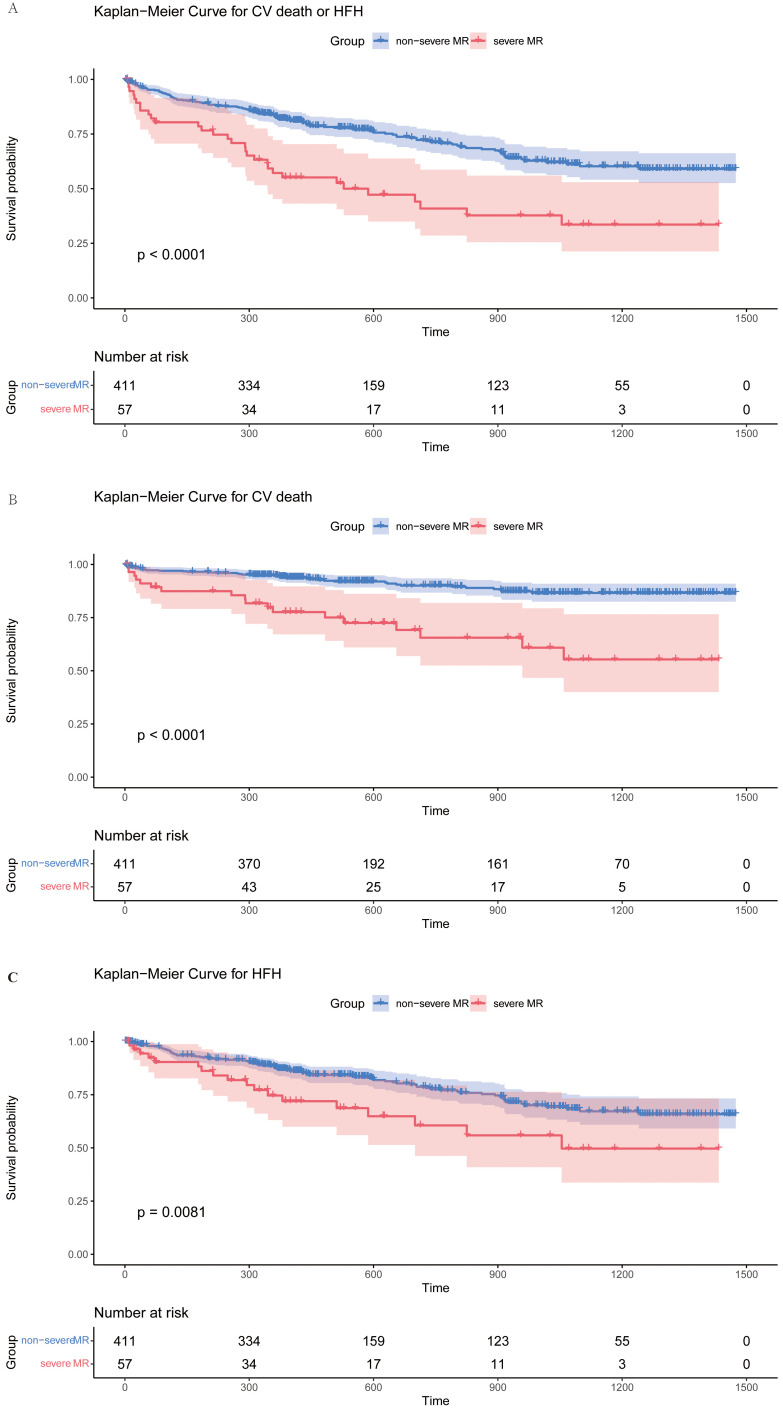
**Accumulated risks for both the primary and secondary endpoints in all patients classified by degrees of MR**. Survival curves for the primary endpoint (A) (log rank *p <* 0.0001), cardiovascular death (B) (log rank *p <* 0.0001) and HFH (C) (log rank *p *= 0.0081) comparing patients with in non-severe and severe MR groups. Log rank test was used to compared the difference between two groups. CV, cardiovascular; HFH, heart failure hospitalization; MR, mitral regurgitation.

The mean follow-up interval for the complete cohort was 578.5 (median = 381–1040) days, with 93.3% of the patients completing the follow-up. In the whole study, 146 (31.2%) of the patients met the primary endpoint, 57 (12.2%) died from cardiovascular events, and 103 (22.0%) were hospitalized for HF. The frequency of the primary endpoint was greater in the severe MR group than in the non-severe group (54.4% *vs* 28.0%; *p <* 0.0001; Fig. 4A). Patients with severe MR exhibited higher risk for a composite outcome of cardiovascular mortality and HFH (HR = 1.88; 95% CI = 1.42–2.31; *p* < 0.001, and adjusted hazard ratio (AHR) = 1.40; 95% CI = 1.03–1.91; *p* = 0.03) (Table 2). The incidence of cardiovascular death (Fig. 4B) and HFH (Fig. 4C) were also greater in the severe MR group. Patients with severe MR had higher risk for cardiovascular mortality (AHR = 1.82; 95% CI = 1.15–2.88; *p *= 0.011; **Supplementary Table 2**). HFH was comparable in the two groups (AHR = 1.17; 95% CI = 0.78–1.77; *p *= 0.44; **Supplementary Table 3**).

**Table 2.** **Predictors of primary endpoints in all patients by univariable and multivariable Cox regression**.

	Univariate analysis		Multivariate analysis	
	HR (95% CI)	*p*	HR (95% CI)	*p*
MR grade (severe *vs* non-severe)	1.88 (1.42–2.31)	<0.001***	1.40 (1.03–1.91)	0.030*
Sex (Female *vs* male)	1.69 (1.15–2.48)	0.007**	1.28 (0.84–1.95)	0.253
Age (per year)	1.01 (1–1.03)	0.102	1 (0.98–1.02)	0.779
Prior atrial fibrillation	1.47 (0.97–2.22)	0.066	1.62 (1.02–2.55)	0.04*
Diabetes mellitus	1.23 (0.89–1.71)	0.21	1.32 (0.92–1.88)	0.134
Hypercholesterolemia	0.88 (0.54–1.43)	0.606	0.84 (0.51–1.39)	0.496
Hypertension	1.17 (0.83–1.65)	0.373	1.01 (0.7–1.47)	0.953
BMI (per kg/$m^{2}$)	0.94 (0.9–0.99)	0.01*	0.97 (0.92–1.03)	0.34
eGFR (per mL/min/1.73 $m^{2}$)	0.98 (0.98–0.99)	<0.001***	0.98 (0.98–0.99)	<0.001***
LVEF (per %)	0.96 (0.94–0.98)	<0.001***	0.96 (0.94–0.98)	<0.001***
Coronary artery disease	1.2 (0.83–1.75)	0.328	1.5 (0.99–2.25)	0.054
Anemia	1.98 (1.21–3.24)	0.007**	1.04 (0.56–1.92)	0.9

BMI, body mass index; eGFR, estimated glomerular filtration rate; LVEF, left ventricular ejection fraction; MR, mitral regurgitation; HR, hazard ratio. * = *p* < 0.05; ** = *p* < 0.01; *** = *p* < 0.001.

### 3.5 Clinical Outcomes in Patients with Distinct ePASP States, Classified by MR Severity

**Fig. 5.**
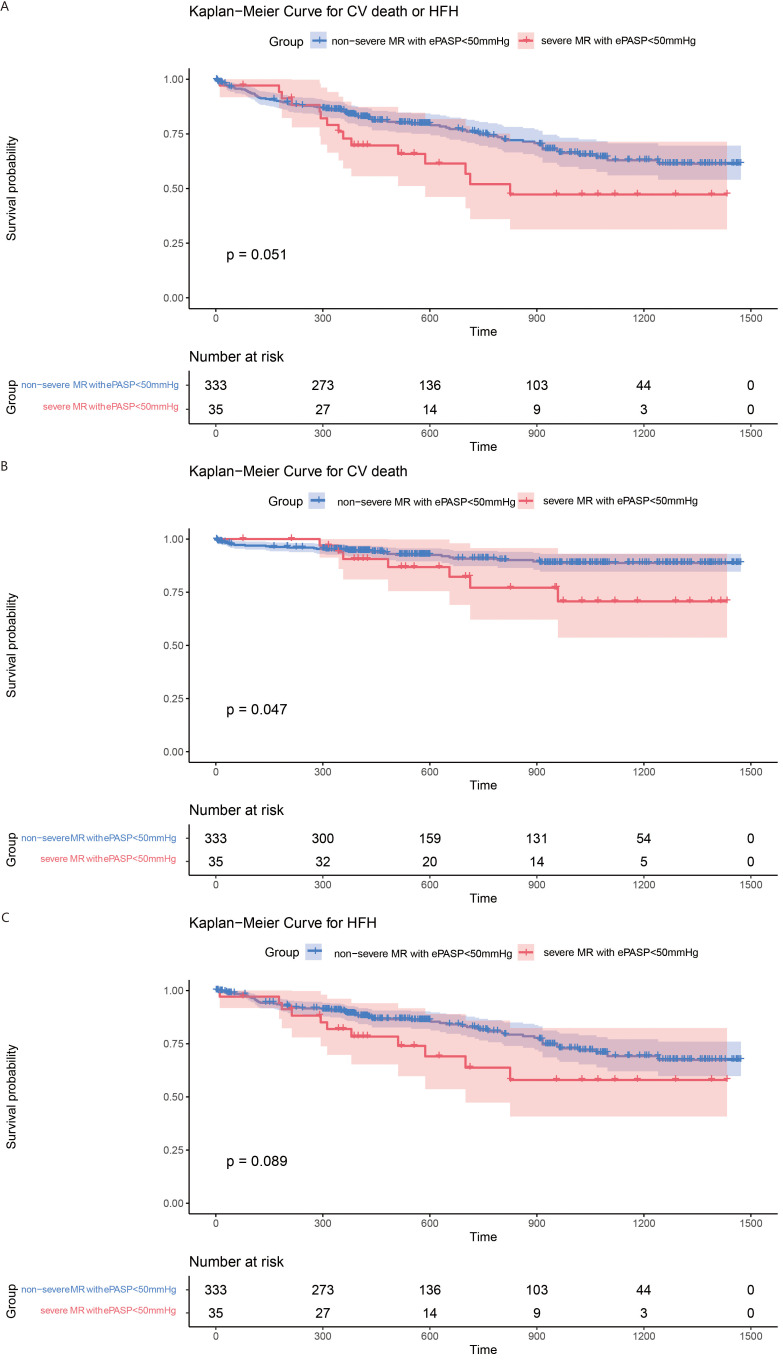
**Accumulated risk for both the primary and secondary endpoints in patients with ePASP <50 mmHg, classified by degrees of MR**. Survival curves comparing patients in non-severe and severe MR groups for (A) the primary endpoint (log rank *p *= 0.051); (B) cardiovascular death (log rank *p *= 0.047); and (C) HFH (log rank *p *= 0.089). CV, cardiovascular; HFH, heart failure hospitalization; MR, mitral regurgitation; ePASP, estimated pulmonary artery systolic pressure.

**Fig. 6.**
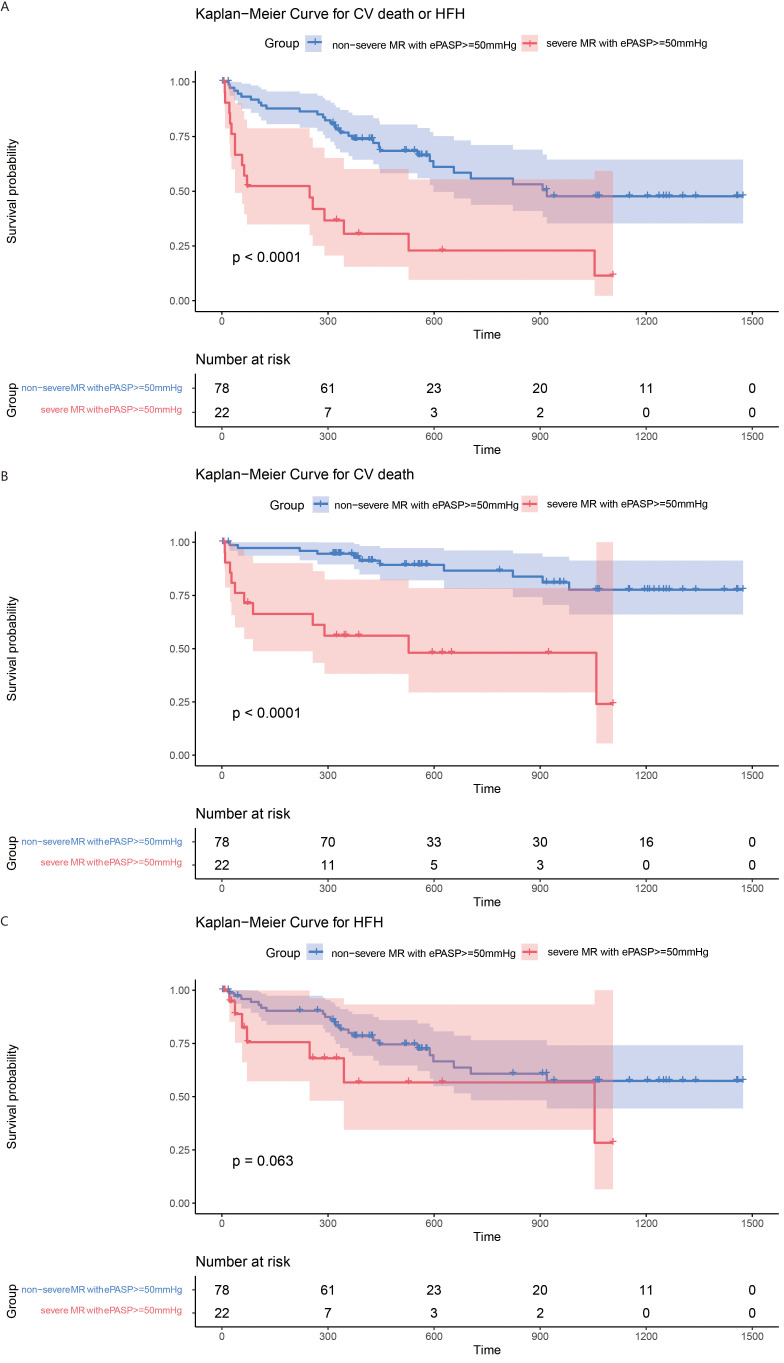
**Accumulated risks for both the primary and secondary endpoints in patients with ePASP ≥50 mmHg, classified by degrees of MR**. Survival curves comparing patients in non-severe and severe MR groups for (A) the primary endpoint (log rank *p* < 0.0001); (B) cardiovascular death (log rank *p* < 0.0001; and (C) HFH (log rank *p* = 0.063). CV, cardiovascular; HFH, heart failure hospitalization; MR, mitral regurgitation; ePASP, estimated pulmonary artery systolic pressure.

The clinical outcome of patients was evaluated with different ePASP levels and stratiﬁed by baseline MR severity. Among patients with ePASP <50 mmHg there was no significant difference for the primary endpoint in the severe MR group (42.9% *vs* 25.5%; *p *= 0.051; Fig. 5A). There was a non-significant “trend” toward increased risk for the primary endpoints in the severe MR group (HR = 1.71; 95% CI = 0.99–2.97; *p *= 0.055; Table 3), but the differences were not statistically significant at this sample size. This “trend”, however, was no longer evident after full adjustments (AHR = 0.99; 95% CI = 0.54–1.84; *p *= 0.98; Table 3). Fig. 6A depicts the frequency of primary endpoints in patients with ePASP ≥50 mmHg (72.7% *vs* 38.5%;* p <* 0.0001). Severe MR was associated with greater risk for primary endpoints (HR = 3.62; 95% CI = 1.96–6.70; *p <* 0.001; Table 4), and the difference remained significant after adjustments (HR = 2.76; 95% CI = 1.28–5.98; *p *= 0.0098; Table 4).

**Table 3.** **Predictors of primary endpoints in patients with ePASP ≤50 mmHg by univariable and multivariable Cox regression**.

	Univariate analysis		Multivariate analysis	
	HR (95% CI)	*p*	HR (95% CI)	*p*
MR grade (severe *vs* non-severe)	1.71 (0.99–2.97)	0.054	0.99 (0.54–1.84)	0.987
Sex (Female *vs* male)	1.57 (0.98–2.5)	0.058	1.21 (0.72–2.01)	0.474
Age (per year)	1.02 (1–1.04)	0.031*	1.00 (0.98–1.03)	0.759
Prior atrial fibrillation	1.55 (0.94–2.56)	0.086	1.68 (0.97–2.9)	0.065
Diabetes mellitus	1.04 (0.69–1.56)	0.843	1.07 (0.69–1.67)	0.752
Hypercholesterolemia	1.14 (0.66–1.98)	0.636	1.36 (0.77–2.42)	0.292
Hypertension	1.36 (0.89–2.07)	0.152	1.26 (0.8–1.99)	0.316
BMI (per kg/$m^{2}$)	0.94 (0.89–1)	0.046*	0.97 (0.9–1.03)	0.304
eGFR (per ml/min/1.73 $m^{2}$)	0.98 (0.97–0.99)	<0.001***	0.98 (0.97–0.99)	<0.001***
LVEF (per %)	0.96 (0.94–0.99)	0.002**	0.96 (0.93–0.98)	<0.001***
Coronary artery disease	1.53 (0.95–2.45)	0.08	1.96 (1.18–3.28)	0.01*
Anemia	2.23 (1.19–4.18)	0.012*	1.08 (0.5–2.31)	0.851

BMI, body mass index; eGFR, estimated glomerular filtration rate; LVEF, left ventricular ejection fraction; MR, mitral regurgitation; ePASP, estimated pulmonary artery systolic pressure; HR, hazard ratio. * = *p* < 0.05; ** = *p* < 0.01; *** = *p* < 0.001.

**Table 4.** **Predictors of primary endpoints in patients with ePASP ≥50 mmHg by univariable and multivariable Cox regression**.

	Univariate analysis		Multivariate analysis	
	HR (95% CI)	*p*	HR (95% CI)	*p*
MR grade (severe *vs* non-severe)	3.62 (1.96–6.7)	<0.001***	2.76 (1.28–5.98)	0.01**
Sex (Female *vs* male)	2.3 (1.15–4.6)	0.018*	2.53 (1.03–6.23)	0.043*
Age (per year)	0.99 (0.97–1.02)	0.507	0.97 (0.94–1.01)	0.129
Prior atrial fibrillation	1.25 (0.6–2.59)	0.55	1.62 (0.67–3.92)	0.281
Diabetes mellitus	1.57 (0.88–2.82)	0.128	2.42 (1.18–4.94)	0.016*
Hypercholesterolemia	0.4 (0.14–1.13)	0.083	0.23 (0.07–0.78)	0.019*
Hypertension	0.72 (0.4–1.31)	0.282	0.47 (0.24–0.96)	0.037*
BMI (per kg/$m^{2}$)	0.93 (0.85–1.02)	0.115	0.97 (0.87–1.09)	0.601
eGFR (per mL/min/1.73 $m^{2}$)	0.99 (0.98–1)	0.091	0.98 (0.97–1)	0.056
LVEF (per %)	0.97 (0.93–1)	0.06	0.96 (0.92–1)	0.062
Coronary artery disease	0.64 (0.35–1.2)	0.163	1.09 (0.51–2.33)	0.83
Anemia	1.22 (0.54–2.72)	0.631	0.64 (0.2–2.03)	0.444

BMI, body mass index; eGFR, estimated glomerular filtration rate; LVEF, left ventricular ejection fraction; MR, mitral regurgitation; ePASP, estimated pulmonary artery systolic pressure; HR, hazard ratio. * = *p* < 0.05; ** = *p* < 0.01; *** = *p* < 0.001.

Patients within the severe MR group with ePASP <50 mmHg exhibited a higher incidence cardiovascular death (Fig. 5B). However, there was no significant difference in cardiovascular mortality risk between the two groups (AHR = 1.34; 95% CI = 0.52–3.48; *p *= 0.54; **Supplementary Table 4**). With respect to patients with ePASP ≥50 mmHg, the frequency of cardiovascular death was considerably greater in the severe MR group than in the non-severe group (Fig. 5B). Patients with severe MR also had an elevated risk of cardiovascular death (AHR = 5.50; 95% CI = 1.88–16.05; *p *= 0.0018; **Supplementary Table 5**) (Fig. 6B). Hospitalization rates and hospital risk for HF were comparable between the two groups regardless of ePASP (Figs. 5C,6C; **Supplementary Tables 6,7**).

## 4. Discussion

The present study examined the prognostic significance of the severity of secondary MR in Chinese HF patients with different levels of ePASP. Our findings indicate that patients with severe secondary MR, especially those with elevated ePASP, face a higher risk of cardiovascular and HF-related mortality. The severity of MR could aid risk categorization of these patients.

Previous studies have shown that patients with HF and secondary MR are negatively affected by elevated ePASP [9]. However, fewer studies have searched for a correlation between secondary MR level and outcomes in HF patients with varying ePASP. Our study builds upon previous findings by revealing that severe secondary MR, at baseline is linked to an increase risk of adverse cardiac outcomes in HF patients, particularly those with elevated ePASP. Moreover, our investigation highlights the higher prognostic value of secondary MR in HF patients with elevated ePASP compared to those without elevated ePASP. In this study, we observed that patients with HF and coexisting PH exhibited a more pronounced impairment of cardiac structure and function compared to those with lower ePASP values. The incorporation of MR severity for further classification accentuated the disparities in cardiac deficits. The dismal outcomes of this subgroup of HF patients may be partially attributed to elevated severity of cardiac impairment. Notably, severe MR emerged as an independent risk factor for the prognosis of HF patients with PH.

The progression of LV remodeling is the primary contributor to PH [13]. Impaired LV filling and diastolic function caused by LV remodeling led to elevated left atrial filling pressure, which is passively transmitted through pulmonary circulation. The pressure elevation in pulmonary circulation produces isolated post-capillary PH [14]. As the condition progresses, the unresolved overload pressure leads to damage and remodeling of veins, capillaries, and small arteries, resulting in combined pre- and post-capillary PH. When coexisting with MR, left atrial pressure rise persists and perpetuates capillary remodeling. PH affects right ventricular function [15]. By ventricular interdependence, a phenomenon where force is transmitted between two ventricles via the myocardium and pericardium, the decreased right ventricular function interferes with LV filling [16]. The worsening of LV loading conditions exacerbates MR. Consequently, the combination of MR and PH creates a detrimental cycle that impacts patients with HF.

Patients with chronic severe secondary MR related to impaired LV systolic function are candidates for transcatheter edge-to-edge repair (TEER), if they also have favorable cardiac structure [8]. The clinical trial Cardiovascular Outcomes Assessment of the MitraClip Percutaneous Therapy for Heart Failure Patients with Functional Mitral Regurgitation (COAPT) provided evidence supporting the favorable benefit of TEER for this subgroup of patients [17]. Furthermore, TEER reduced ePASP elevation in patients with severe MR 30 days later; even a 5 mmHg drop in ePASP was related to improved clinical outcomes [9]. Notably, patients with ePASP ≥70 mmHg were excluded from the COAPT trial, and the median ePASP in that trial was 43.1 mmHg (less than 50 mmHg) as measured by echocardiography. Therefore, the effect of TEER on decreasing ePASP in HF patients with exacerbating PH remained unknown. In addition, the trial evaluated the short-term effects of TEER on ePASP; long-term investigation is required for determining whether the change in ePASP was sustainable. As previously demonstrated, increases in ePASP are indicative of progressive cardiac dysfunction. The MITRA-FA trial (Percutaneous Repair with the MitraClip Device for Severe Functional/Secondary Mitral Regurgitation) [18] indicates that patients with significant LV remodeling are less likely to derive clinical benefit from TEER compared to MR level. Consequently, despite the high-risk nature of patients with both PH and severe MR, the impact of TEER on PH and overall outcomes in this patient group remains a topic of debate.

Our study had several limitations. First, it was an observational study conducted at a single location, thus uncontrolled variables may have affected the results. Generalization of the finding to the entire population of HF patients with PH should be approached with caution due to the small sample size assigned to the severe MR group (57 *vs* 411 for the non-severe MR group). Second, PASP was determined using transthoracic echocardiography rather than by RHC. Although there is a moderate correlation between RHC and echocardiographic measurements, right heart catheterization is known to provide additional information, including the classification of subtypes of PH based on hemodynamic measurements, which could affect the outcomes [19]. Even in cases of severe LV dysfunction, it remains challenging to completely exclude non-cardiac causes of PH without confirmation through a RHC. Third, the treatment of HF in some cases might be inadequate; for instance, only 3.8% of patients in the cohort received CRT or ICD, which is below the rates reported in previous studies [17]. Fourth, few patients underwent mitral valve intervention during hospitalization and follow-up because the study was conducted before the updates of guidelines for the managements of patients with secondary severe MR related with HF.

## 5. Conclusions

he present study highlighted the prognostic significance of secondary MR for death from cardiovascular events or HFH in HF patients. Secondary MR has a notable prognostic value in patients with elevated ePASP, which is diminished among patients with relatively lower ePASP.

## Data Availability

The data sets generated and analyzed during the current study are not publicly available due to their containing information that could compromise the privacy of research participants, but are available from the corresponding author on reasonable request.

## Abbreviations

ACEI, angiotensin-converting enzyme inhibitor; AHR, adjusted hazard ratio; ARB, angiotensin receptor blocker; ARNI, Angiotensin receptor/neprilysin dual inhibitors; BMI, body mass index; CI, confidential interval; COAPT, Cardiovascular Outcomes Assessment of the MitraClip Percutaneous Therapy for Heart Failure Patients with Functional Mitral Regurgitation; CRT, cardiac resynchronization therapy; CV, cardiovascular; eGFR, estimated glomerular filtration rate; EROA, effective regurgitant oriﬁce area; HR, hazard ratio; HFH, heart failure hospitalization; ICD, implantable cardioverter-defibrillator; LV, left ventricular; LVEF, left ventricular ejection fraction; LVEDD, LV end-diastolic dimension; LVESD, LV end-systolic dimension; LAD, left atrial dimension; MITRA-FA, Percutaneous Repair with the MitraClip Device for Severe Functional/Secondary Mitral Regurgitation; MR, mitral regurgitation; MRA, mineralocorticoid receptor antagonist; NT-proBNP, N-terminal pro–B-type natriuretic peptide; PASP, pulmonary artery systolic pressure; PCI, percutaneous coronary intervention; PISA, proximal isovelocity surface area; RHC, right heart catheterization; RV, regurgitant volume; TEER, transcatheter edge-to-edge repair; TR, tricuspid regurgitation; VCW, vena contracta width.

## Supplementary Material

Supplementary material associated with this article can be found, in the online version, at https://doi.org/10.31083/j.rcm2411316.
