# Supplementary material for: The Prognostic Significance of Secondary Mitral Regurgitation in Heart Failure Patients with Varying Estimated Pulmonary Artery Systolic Pressure
Source: Rev Cardiovasc Med. 2023 Nov 16;24(11):316. doi: 10.31083/j.rcm2411316 (PMC11272836; doi:10.31083/j.rcm2411316)
Supplement: Supplementary file 1 [file 2153-8174-24-11-316-s1.docx]

**Supplementary Table 1. The Schoenfeld residual test and variance inflation factor of the variables in multivariable Cox regression model for primary endpoints.**

| Variable | Schoenfeld residual test *p* | Variance inflation factor |
| --- | --- | --- |
| MR grade (severe vs non-severe) | 0.2 | 1.17 |
| Sex (Female vs male) | 0.23 | 1.14 |
| Age (per year) | 0.66 | 1.55 |
| Prior atrial fibrillation | 0.12 | 1.13 |
| diabetes mellitus | 0.74 | 1.11 |
| hypercholesterolemia | 0.79 | 1.05 |
| Hypertension | 0.17 | 1.16 |
| BMI (per kg/m2) | 0.98 | 1.29 |
| eGFR (per ml/min/1.73 m2) | 0.08 | 1.34 |
| LVEF (per %) | 0.79 | 1.20 |
| coronary artery disease | 0.64 | 1.23 |
| Anemia | 0.28 | 1.28 |
| global | 0.17 | / |

BMI, body mass index; eGFR, estimated glomerular filtration rate; LVEF, left ventricular ejection fraction; MR, mitral regurgitation.

**Supplementary Table 2. Predictors of cardiovascular death in all patients by univariable and multivariable Cox regression.**

|  | Univariate analysis | | Multivariate analysis | |
| --- | --- | --- | --- | --- |
|  | HR (95% CI) | *p* | HR (95% CI) | *p* |
| MR grade (severe vs non-severe) | 2.57 (1.73, 3.81) | <0.001 | 1.82(1.15,2.88) | 0.011 |
| Sex (Female vs male) | 1.99 (1.1, 3.6) | 0.022 | 1(0.52,1.93) | 0.998 |
| Age (per year) | 1.03 (1.01, 1.06) | 0.012 | 1.01(0.98,1.04) | 0.61 |
| Prior atrial fibrillation | 1.65 (0.89, 3.07) | 0.111 | 2.14(1.05,4.34) | 0.035 |
| diabetes mellitus | 1.21 (0.71, 2.04) | 0.485 | 1.22(0.68,2.19) | 0.503 |
| hypercholesterolemia | 0.81 (0.37, 1.79) | 0.601 | 0.74(0.32,1.7) | 0.473 |
| Hypertension | 1.21 (0.7, 2.1) | 0.494 | 0.88(0.48,1.63) | 0.684 |
| BMI (per kg/m^2^) | 0.88 (0.81, 0.95) | 0.001 | 0.95(0.86,1.05) | 0.316 |
| eGFR (per ml/min/1.73 m^2^) | 0.97 (0.96, 0.98) | <0.001 | 0.97(0.96,0.98) | <0.001 |
| LVEF (per %) | 0.97 (0.94, 0.99) | 0.021 | 0.97(0.93,1) | 0.048 |
| coronary artery disease | 1.37 (0.74, 2.54) | 0.32 | 1.51(0.76,2.98) | 0.24 |
| Anemia | 3.62 (1.87, 7.01) | <0.001 | 1.34(0.58,3.07) | 0.493 |

BMI, body mass index; eGFR, estimated glomerular filtration rate; LVEF, left ventricular ejection fraction; MR, mitral regurgitation.

* = *p* < 0.05; ** = *p* < 0.01; *** = *p* < 0.001.

**Supplementary Table 3. Predictors of heart failure hospitalization in all patients by univariable and multivariable Cox regression.**

|  | Univariate analysis | | Multivariate analysis | |
| --- | --- | --- | --- | --- |
|  | HR (95% CI) | *p* | HR (95% CI) | *p* |
| MR grade (severe vs non-severe) | 1.61 (1.12, 2.31) | 0.009 | 1.17(0.78,1.77) | 0.439 |
| Sex (Female vs male) | 1.62 (1.02, 2.58) | 0.041 | 1.6(0.97,2.64) | 0.068 |
| Age (per year) | 1 (0.99, 1.02) | 0.733 | 0.99(0.97,1.01) | 0.491 |
| Prior atrial fibrillation | 1.34 (0.8, 2.23) | 0.263 | 1.4(0.8,2.45) | 0.243 |
| diabetes mellitus | 1.14 (0.77, 1.69) | 0.51 | 1.23(0.81,1.88) | 0.338 |
| hypercholesterolemia | 1.09 (0.64, 1.86) | 0.747 | 1.05(0.6,1.82) | 0.864 |
| Hypertension | 1.07 (0.72, 1.61) | 0.725 | 1.05(0.68,1.61) | 0.84 |
| BMI (per kg/m^2^) | 0.97 (0.91, 1.02) | 0.245 | 0.98(0.92,1.05) | 0.609 |
| eGFR (per ml/min/1.73 m^2^) | 0.99 (0.98, 1) | 0.027 | 0.99(0.98,1) | 0.066 |
| LVEF (per %) | 0.96 (0.94, 0.98) | 0.001 | 0.96(0.94,0.98) | 0.001 |
| coronary artery disease | 1.21 (0.77, 1.89) | 0.404 | 1.61(0.99,2.62) | 0.054 |
| Anemia | 1.03 (0.48, 2.22) | 0.938 | 0.67(0.27,1.63) | 0.375 |

BMI, body mass index; eGFR, estimated glomerular filtration rate; LVEF, left ventricular ejection fraction; MR, mitral regurgitation.

* = *p* < 0.05; ** = *p* < 0.01; *** = *p* < 0.001.

**Supplementary Table 4. Predictors of cardiovascular death in patients with PASP≤50mmHg by univariable and multivariable Cox regression.**

BMI, body mass index; eGFR, estimated glomerular filtration rate; LVEF, left ventricular ejection fraction; MR, mitral regurgitation.

* = *p* < 0.05; ** = *p* < 0.01; *** = *p* < 0.001.

|  | Univariate analysis | | Multivariate analysis | |
| --- | --- | --- | --- | --- |
|  | HR (95% CI) | *p* | HR (95% CI) | *p* |
| MR grade (severe vs non-severe) | 2.27 (0.99, 5.19) | 0.053 | 1.34(0.52,3.48) | 0.541 |
| Sex (Female vs male) | 2.13 (1.02, 4.44) | 0.043 | 1.2(0.51,2.83) | 0.67 |
| Age (per year) | 1.03 (1, 1.06) | 0.048 | 1(0.97,1.04) | 0.821 |
| Prior atrial fibrillation | 1.41 (0.62, 3.23) | 0.418 | 1.8(0.72,4.54) | 0.211 |
| diabetes mellitus | 0.92 (0.46, 1.85) | 0.819 | 0.81(0.37,1.79) | 0.606 |
| hypercholesterolemia | 1.28 (0.53, 3.09) | 0.582 | 1.81(0.7,4.67) | 0.221 |
| Hypertension | 1.6 (0.77, 3.32) | 0.211 | 1.23(0.55,2.75) | 0.616 |
| BMI (per kg/m^2^) | 0.9 (0.82, 1) | 0.042 | 0.96(0.85,1.08) | 0.465 |
| eGFR (per ml/min/1.73 m^2^) | 0.96 (0.95, 0.98) | <0.001 | 0.97(0.95,0.99) | <0.001 |
| LVEF (per %) | 0.98 (0.94, 1.02) | 0.298 | 0.97(0.93,1.02) | 0.204 |
| coronary artery disease | 1.71 (0.75, 3.91) | 0.205 | 2.09(0.83,5.24) | 0.117 |
| Anemia | 4.85 (2.11, 11.12) | <0.001 | 1.56(0.52,4.67) | 0.425 |

**Supplementary Table 5. Predictors of cardiovascular death in patients with PASP>50mmHg by univariable and multivariable Cox regression.**

|  | Univariate analysis | | Multivariate analysis | |
| --- | --- | --- | --- | --- |
|  | HR (95% CI) | *p* | HR (95% CI) | *p* |
| MR grade (severe vs non-severe) | 5.93 (2.54, 13.87) | <0.001 | 5.5(1.88,16.05) | 0.002 |
| Sex (Female vs male) | 2.07 (0.74, 5.78) | 0.163 | 1.61(0.38,6.72) | 0.515 |
| Age (per year) | 1.03 (0.99, 1.07) | 0.175 | 1.01(0.94,1.07) | 0.843 |
| Prior atrial fibrillation | 2.01 (0.78, 5.14) | 0.147 | 2.28(0.65,8.02) | 0.198 |
| diabetes mellitus | 1.5 (0.65, 3.47) | 0.346 | 1.66(0.65,4.26) | 0.288 |
| hypercholesterolemia | 0.22 (0.03, 1.64) | 0.14 | 0.07(0.01,0.72) | 0.026 |
| Hypertension | 0.71 (0.3, 1.66) | 0.426 | 0.44(0.15,1.28) | 0.131 |
| BMI (per kg/m^2^) | 0.85 (0.75, 0.96) | 0.009 | 0.87(0.7,1.08) | 0.216 |
| eGFR (per ml/min/1.73 m^2^) | 0.98 (0.96, 0.99) | 0.002 | 0.96(0.94,0.98) | 0.001 |
| LVEF (per %) | 0.96 (0.91, 1.01) | 0.091 | 0.94(0.88,1.01) | 0.109 |
| coronary artery disease | 0.9 (0.35, 2.31) | 0.834 | 1.31(0.42,4.15) | 0.642 |
| Anemia | 1.74 (0.59, 5.16) | 0.318 | 0.85(0.18,3.98) | 0.839 |

BMI, body mass index; eGFR, estimated glomerular filtration rate; LVEF, left ventricular ejection fraction; MR, mitral regurgitation.

* = *p* < 0.05; ** = *p* < 0.01; *** = *p* < 0.001.

**Supplementary Table 6. Predictors of heart failure hospitalization in patients with PASP≤50mmHg by univariable and multivariable Cox regression.**

|  | Univariate analysis | | Multivariate analysis | |
| --- | --- | --- | --- | --- |
|  | HR (95% CI) | *p* | HR (95% CI) | *p* |
| MR grade (severe vs non-severe) | 1.73 (0.91, 3.29) | 0.094 | 1.06(0.51,2.19) | 0.874 |
| Sex (Female vs male) | 1.61 (0.93, 2.77) | 0.087 | 1.5(0.83,2.69) | 0.18 |
| Age (per year) | 1.02 (0.99, 1.04) | 0.166 | 1(0.98,1.03) | 0.736 |
| Prior atrial fibrillation | 1.61 (0.9, 2.88) | 0.112 | 1.53(0.8,2.94) | 0.197 |
| diabetes mellitus | 0.98 (0.61, 1.58) | 0.926 | 1.03(0.62,1.72) | 0.909 |
| hypercholesterolemia | 1.3 (0.7, 2.41) | 0.414 | 1.47(0.77,2.8) | 0.247 |
| Hypertension | 1.14 (0.7, 1.84) | 0.599 | 1.14(0.68,1.91) | 0.614 |
| BMI (per kg/m^2^) | 0.97 (0.91, 1.04) | 0.36 | 0.99(0.92,1.07) | 0.782 |
| eGFR (per ml/min/1.73 m^2^) | 0.99 (0.98, 1) | 0.025 | 0.99(0.98,1) | 0.063 |
| LVEF (per %) | 0.96 (0.94, 0.99) | 0.007 | 0.96(0.93,0.99) | 0.004 |
| coronary artery disease | 1.52 (0.87, 2.65) | 0.138 | 2.04(1.12,3.72) | 0.02 |
| Anemia | 1.05 (0.38, 2.87) | 0.93 | 0.61(0.2,1.92) | 0.402 |

BMI, body mass index; eGFR, estimated glomerular filtration rate; LVEF, left ventricular ejection fraction; MR, mitral regurgitation.

* = *p* < 0.05; ** = *p* < 0.01; *** = *p* < 0.001.

**Supplementary Table 7. Predictors of heart failure hospitalization in patients with PASP>50mmHg by univariable and multivariable Cox regression.**

|  | Univariate analysis | | Multivariate analysis | |
| --- | --- | --- | --- | --- |
|  | HR (95% CI) | *p* | HR (95% CI) | *p* |
| MR grade (severe vs non-severe) | 2.2 (0.94, 5.18) | 0.07 | 1.3(0.47,3.56) | 0.615 |
| Sex (Female vs male) | 1.93 (0.77, 4.8) | 0.158 | 3.1(0.94,10.18) | 0.062 |
| Age (per year) | 0.97 (0.95, 1) | 0.066 | 0.96(0.92,1) | 0.039 |
| Prior atrial fibrillation | 0.81 (0.28, 2.32) | 0.692 | 1.08(0.33,3.57) | 0.899 |
| diabetes mellitus | 1.49 (0.72, 3.06) | 0.28 | 3.18(1.22,8.26) | 0.018 |
| hypercholesterolemia | 0.64 (0.22, 1.84) | 0.406 | 0.49(0.15,1.66) | 0.254 |
| Hypertension | 0.79 (0.38, 1.67) | 0.545 | 0.59(0.25,1.38) | 0.225 |
| BMI (per kg/m^2^) | 0.96 (0.86, 1.07) | 0.492 | 0.95(0.83,1.07) | 0.372 |
| eGFR (per ml/min/1.73 m^2^) | 1 (0.99, 1.02) | 0.902 | 1(0.98,1.02) | 0.905 |
| LVEF (per %) | 0.97 (0.93, 1.01) | 0.124 | 0.96(0.91,1.01) | 0.147 |
| coronary artery disease | 0.62 (0.29, 1.32) | 0.216 | 0.75(0.3,1.88) | 0.541 |
| Anemia | 0.75 (0.23, 2.48) | 0.64 | 0.42(0.09,2.1) | 0.293 |

BMI, body mass index; eGFR, estimated glomerular filtration rate; LVEF, left ventricular ejection fraction; MR, mitral regurgitation.

* = *p* < 0.05; ** = *p* < 0.01; *** = *p* < 0.001.
